# Association between polymorphisms in the *APOB* gene and hyperlipidemia in the Chinese Yugur population

**DOI:** 10.1590/1414-431X20176613

**Published:** 2017-09-12

**Authors:** Q-L. Gu, Y. Han, Y-M. Lan, Y. Li, W. Kou, Y-S. Zhou, X-J. Hai, B. Yan, C-H. Ci

**Affiliations:** 1 The Institute of Minority Physique and Health, Medical College of Northwest University for Nationalities, Lanzhou, China; 2 College of Life Science and Technology, Huazhong University of Science and Technology, WuHan, China; 3 The Institute of Medical Genetics, School of Basic Medical Sciences, Lanzhou University, Lanzhou, China

**Keywords:** Yugur minority, Apolipoprotein B gene, Single nucleotide polymorphism, Hyperlipidemia, Serum lipids

## Abstract

We investigated the influence of apolipoprotein B gene (*APOB*) variants on the risk of hyperlipidemia (HL) in 631 middle-aged and elderly members of the Chinese Yugur population (HL, n=336; normolipidemia, n=295). *APOB* polymorphisms were identified using mass spectrometry, and five single nucleotide polymorphisms (rs1042034, rs2163204, rs512535, rs676210, and rs679899) and serum lipids were further analyzed. rs1042034 and rs676210 were significantly associated with HL (P*<*0.05). Compared with the GG or AA genotype, individuals with AG and AG+AA in rs1042034 and with AG and AG+GG in rs676210 had a 1.67-fold (95%CI=1.20–2.33),1.63-fold (95%CI=1.19–2.24), 1.72-fold (95%CI=1.24–2.40), and 1.67-fold (95%CI=1.21–2.291) increased risk of high HL, respectively. rs2163204 was in strong linkage disequilibrium with rs1042034, rs676210, and rs679899, and strong disequilibrium was observed between rs1042034 and rs676210 (D′>0.9). Compared with the GTGAA haplotype, haplotypes ATGGA and ATAGG were more strongly associated with HL [odds ratio (OR)=1.46, 95%CI=0.02–2.11; OR=1.63, 95%CI=1.03–2.60, respectively]. The risk factors age (P*=*0.008), body mass index (P*<*0.0001), GA+GG genotype in rs676210 (P*=*0.009), and alcohol consumption (P*=*0.056) contributed strongly to HL development. The A allele of rs1042034 and the G allele of rs676210 may thus predispose middle-aged and elderly members of the Chinese Yugur population to HL in combination with other genetic or nutritional factors, and could be used as new genetic markers for HL screening.

## Introduction

Hyperlipidemia (HL) is a polygenic disease triggered by a combination of genetic and environmental factors. HL is also a well-established risk factor of atherosclerosis, coronary heart disease, cerebral infarction, and hypertension (1–5), all of which are threats to public health. The incidence of HL has gradually increased, leading to renewed research interest in this condition.

Apolipoprotein B (APOB) is a major structural protein of chylomicron, a very-low-density lipoprotein, and low-density lipoprotein cholesterol (LDLc). Thus far, two subunits of APOB, APOB100 and APOB48, have been discovered, and APOB100 is the main component of LDL; indeed, APOB comprises ∼90% of LDL (6). The *APOB* gene, which is located on human chromosome 2p23-24, is 43 kb in length and contains 28 introns and 29 exons (7). The structure of this gene differs from other apolipoprotein genes, and multiple genetic mutations in the gene have been identified. Notably, mutations in the *APOB* gene may lead to elevated LDL levels and other lipid metabolic disorders as well as an increased risk of coronary heart disease owing to higher levels of serum APOB, even in the presence of normal levels of LDL (8-10). Therefore, the *APOB* gene has become a major focus for many researchers.

Several studies have shown that race, region, and disease state are associated with variations in the distributions of APOB isoforms. In addition, many studies have examined polymorphisms in the *APOB* gene in populations of different races and regions (11–15). For example, Kim et al. (16) found that the single nucleotide polymorphism (SNP) rs1042034 in the *APOB* gene increased serum total cholesterol (TC) levels in Americans, and Kulminski et al. (17) found that the CC genotype of SNP rs1042034 was significantly associated with high serum TC levels in younger patients with cardiovascular diseases in America, but acted as a protective factor in elderly individuals. However, Ou et al. (18) reported that the SNP rs1042034 is not associated with HL in Chinese Han individuals living in the Shihezi area of Xinjiang.

China has 56 ethnic groups; the Han Chinese comprise the largest ethnic group, whereas individuals of the Yugur ethnicity are a rare minority and the oldest nomad population in China, with only 14,378 individuals (19). The Yugur population represents a unique minority in Gansu province, and most members of this population live in the Gansu Sunan district. The region is located in the hinterlands of the Qilian Mountains, which has an average elevation of 2500 m. The low population mobility and high probability of marriage within the race have led to a similar genetic background among individuals of this ethnic group.

Furthermore, the Yugur population has maintained a nomadic lifestyle and eating habits, and thus the Yugur lifestyle is largely based on animal husbandry. In our previous studies (20,21), we found that individuals of the Yugur ethnicity have a higher prevalence of dyslipidemia than the Han Chinese living in the same region. Notably, these two groups share similar eating habits, suggesting a genetic component contributing to the increased incidence of dyslipidemia in Yugurs. However, this hypothesis has not yet been directly investigated.

Most SNP analyses focus on evaluating whether individual SNPs independently affect the risk of disease, ignoring the effects of interactions among multiple SNPs and linkage disequilibrium (LD) on the disease. In fact, this synergistic effect may play a role in the pathogenic characteristics of a gene. For instance, previous studies (22,23) showed that many haplotypes can increase the individual disease risk.

Therefore, we aimed to investigate the independent and joint effects of five common variants (rs1042034, rs2163204, rs512535, rs676210, and rs679899) of the *APOB* gene on the risk of HL in the Yugur population. Our results should provide important insights into the role of genetic variants in determining the risk of HL.

## Material and Methods

### Study participants

The study protocol was approved by the Medical Ethics Committee of the Northwest University for Nationalities. All participants signed informed consent forms prior to enrolment. In total, 631 unrelated male and female individuals of the Yugur population, within an age range of 45-80 years (mean 53 years), were enrolled from stratified randomized cluster samples. All individuals were healthy and permanent residents of the Sunan region, representing at least three Yugur generations. The participation rate among eligible subjects was 88%.

### Epidemiological survey

Systolic and diastolic blood pressure (SBP and DBP, respectively) values were measured. Individuals were asked to sit quietly for 30 min without having tea, coffee, or cigarettes before measurement of SBP and DBP. BP was measured three times at intervals of 5 min, and the average value of three measurements was used. Hypertension was diagnosed when the SBP and DBP were equal to or greater than 140 and 90 mmHg, respectively, and were confirmed by three tests on different days. Blood glucose, TC, triglycerides (TGs), high-density lipoprotein cholesterol (HDLc), and LDLc were evaluated using an automatic biochemical meter (Olympus 2007, Japan), and with a blood lipid kit (JianCheng Bioengineering Institute, China) per the manufacturer instructions.

Diabetes mellitus was diagnosed per the criteria of the American Diabetes Association (taking hypoglycemic agents, fasting serum glucose level ≥7.0 mM, or a 2-h postprandial glucose level ≥11.1 mM in two measurements). In total, 336 participants were included in the HL group, and 295 participants were included in the normolipidemia group according to their serum lipid results. Individuals with a fasting TC value ≥6.22 mM, TGs ≥2.26 mM, HDLc<1.04 mM, and/or LDLc>4.14 mM were included in the hyperlipidemic group. Individuals with a fasting TC value<5.18 mM, TGs<1.70 mM, HDLc ≥1.04 mM, and/or LDLc<3.37 mM were included in the normolipidemic group (24).

Smokers were defined as those smoking more than 5 cigarettes daily (25); an alcohol consumer was defined as an individual with a weekly alcohol intake of more than 140 g for men or more than 70 g for women (26), based on self-reporting. Body mass index (BMI; kg/m^2^) was measured using standard methods and instruments.

### Genetic analyses

DNA was isolated from blood samples collected in ethylenediaminetetraacetic acid tubes using standard procedures using a blood genomic DNA extraction kit (centrifugal column type; Tiangen Biotech,China), and the concentration of DNA was determined using a spectrophotometer (Persee, China) at the Medicine Central Laboratory of the Northwest University for Nationalities. Based on a literature search, we selected the following five polymorphisms in the *APOB* gene that had been reported to have a mutation frequency greater than 5% and that were associated with alterations in lipid metabolism in our study cohort: rs1042034 (G>A), rs2163204 (T>G), rs512535 (G>A), rs676210 (A>G), and rs679899 (A>G). These polymorphisms were analyzed using matrix-assisted laser desorption ionization time-of-flight mass spectrometry (MALDI-TOF-MS) at Shenzhen Huada Gene Research Institute, using the MS-Sequenom option to detect SNPs. Each sample was amplified by the multiple polymerase chain reaction (PCR) technique. Shrimp alkaline phosphatase was used to digest the multiple residual dNTPs and join extension primers (next to the SNP target locus) using ddNTP (ddNTP Set, 20 mM solutions, CELLutions Biosystems Inc., Canada,) single-base outspread, and the extension primers and extended products were detected with MALDI-TOF MassARRAY system (Sequenom Inc., USA,). SNP genotyping was performed based on molecular weight analysis.

### Statistical analyses

All statistical analyses were performed using the statistical software package SPSS19.0 (USA). Linear regression analysis was performed with adjustment for age and gender. Differences in mean values were assessed using unpaired *t*-tests. Categorical variables were compared using chi-square tests. Allele and genotype frequencies of the SNPs were calculated and tested for departures from Hardy-Weinberg equilibrium using chi-square tests. Dominant and recessive models were constructed for the rare allele of each polymorphism, and univariate odds ratios (ORs) with 95% confidence intervals (CIs) were calculated. One-way analysis and multivariate regression were performed for HL risk factor analysis. Linkage and haplotype analyses were performed using SHEsis genetic-statistical software (http://analysis.bio-x.cn/SHEsisMain.htm). Specifically, standardized coefficient of LD (|D′|) values closer to 1 indicated a higher degree of LD. In addition, Pearson's correlation coefficients (r values) between the alleles of two loci were calculated, with associated P values. Results with P*<*0.05 were considered to be statistically significant.

## Results

### General characteristics, serum lipid levels, and SNP frequencies

There was no significant difference in gender between the HL group (152 men and 143 women) and normolipidemia group (121 men and 174 women; P*=*0.286). However, the mean age, BMI, SBP, DBP, TC, TGs, HDLc, LDLc, blood glucose, cigarette smoking habit, and drinking habit differed significantly between the two study groups (P<0.05; Table 1).

**Table 1.** Characteristics of the study population.

	Normolipidemia (n=295)	Hyperlipidemia (n=336)	P-value
Male gender (%)	41.02	45.24	0.286
Age (years)	52.31±8.83	54.24±9.35	0.008
BMI (kg/m^2^)	23.24±2.61	24.37±3.15	<0.001
SBP (mmHg)	117.07±10.61	118.77±10.51	0.044
DBP (mmHg)	77.87±6.18	78.64±5.36	0.018
Total cholesterol (mM)	3.52±0.77	4.25±1.24	<0.001
Triglycerides (mM)	1.24±0.43	2.36±1.33	<0.001
HDLc (mM)	1.22±0.23	1.09±0.42	<0.001
LDLc (mM)	1.87±0.62	2.12±0.93	<0.001
Blood glucose (mM)	4.62±1.06	4.74±1.14	0.009
Smoker (%)	23.05	27.98	0.005
Drinking (%)	23.39	31.25	0.006

Data are reported as means±SD or percentage. BMI: body mass index; SBP: systolic blood pressure; DBP: diastolic blood pressure; HDLc: high-density lipoprotein cholesterol; LDLc: low-density lipoprotein cholesterol. Statistical analysis was carried out with Student's *t*-test and the chi-square test.

SNPs were compatible with Hardy-Weinberg expectations (all P>0.05; Table 2). Genotype and allele frequencies were calculated and compared to identify differences between the normolipidemia and HL groups. The frequencies of the analyzed SNPs rs1042034 and rs676210 showed significant differences between the two groups (P*=*0.009; P*=*0.005). The other three SNPs (rs2163204, rs512535, and rs679899) showed no significant differences between the two groups (Table 2). These findings suggested that the effects of SNPs rs1042034 and rs676210 on blood lipids in the Yugur population might be greater than those of the three other SNPs reported previously.

**Table 2.** Genotypic associations of five SNPs with hyperlipidemia.

SNP	Model	Genotype	Controls	Cases	OR (95%CI)	P-value
rs1042034	–	–	n=295	n=335	–	–
	P(HWE)	–	0.275	0.219	–	–
	Co-dominant	G/G	175 (59.3%)	158 (47.2%)	1	0.009
		A/G	100 (33.9%)	151 (45.1%)	1.67 (1.20–2.33)	
		A/A	20 (6.8%)	26 (7.8%)	1.44 (0.77–2.68)	
	Dominant	G/G	175 (59.3%)	158 (47.2%)	1	0.002
		A/G-A/A	120 (40.7%)	177 (52.8%)	1.63 (1.19–2.24)	
	Recessive	G/G-A/G	275 (93.2%)	309 (92.2%)	1	0.64
		A/A	20 (6.8%)	26 (7.8%)	1.16 (0.63–2.12)	
rs2163204	–	–	n=295	n=336	–	–
	P(HWE)	–	0.100	0.210	–	–
	Co-dominant	T/T	238 (80.7%)	274 (81.5%)	1	0.50
		G/T	57 (19.3%)	61 (18.1%)	0.93 (0.62–1.39)	
		G/G	0 (0%)	1 (0.3%)	NA (0.00-NA)	
	Dominant	T/T	238 (80.7%)	274 (81.5%)	1	0.78
		G/T-G/G	57 (19.3%)	62 (18.4%)	0.94 (0.63–1.41)	
	Recessive	T/T-G/T	295 (100%)	335 (99.7%)	1	0.26
		G/G	0 (0%)	1 (0.3%)	NA (0.00-NA)	
rs512535	–	–	n=295	n=336	–	–
	P(HWE)	–	0.530	0.566	–	–
	Co-dominant	G/G	151 (51.2%)	180 (53.6%)	1	0.79
		A/G	117 (39.7%)	129 (38.4%)	0.92 (0.66–1.29)	
		A/A	27 (9.2%)	27 (8%)	0.84 (0.47–1.49)	
	Dominant	G/G	151 (51.2%)	180 (53.6%)	1	0.55
		A/G-A/A	144 (48.8%)	156 (46.4%)	0.91 (0.66–1.24)	
	Recessive	G/G-A/G	268 (90.8%)	309 (92%)	1	0.62
		A/A	27 (9.2%)	27 (8%)	0.87 (0.50–1.52)	
rs676210		–	n=295	n=336	–	–
	P(HWE)	–	0.241	0.143	–	–
	Co-dominant	A/A	176 (59.7%)	158 (47%)	1	0.005
		G/A	99 (33.6%)	153 (45.5%)	1.72 (1.24–2.40)	
		G/G	20 (6.8%)	25 (7.4%)	1.39 (0.74–2.60)	
	Dominant	A/A	176 (59.7%)	158 (47%)	1	0.002
		G/A-G/G	119 (40.3%)	178 (53%)	1.67 (1.21–2.29)	
	Recessive	A/A-G/A	275 (93.2%)	311 (92.6%)	1	0.75
		G/G	20 (6.8%)	25 (7.4%)	1.11 (0.60–2.03)	
rs679899	–	–	n=295	n=336	–	–
	P(HWE)	–	0.329	0.936	–	–
	Co-dominant	A/A	197 (66.8%)	214 (63.7%)	1	0.66
		G/A	85 (28.8%)	108 (32.1%)	1.17 (0.83–1.65)	
		G/G	13 (4.4%)	14 (4.2%)	0.99 (0.45–2.16)	
	Dominant	A/A	197 (66.8%)	214 (63.7%)	1	0.42
		G/A-G/G	98 (33.2%)	122 (36.3%)	1.15 (0.82–1.59)	
	Recessive	A/A-G/A	282 (95.6%)	322 (95.8%)	1	0.88
		G/G	13 (4.4%)	14 (4.2%)	0.94 (0.44–2.04)	

SNP: single nucleotide polymorphism; OR: odds ratio; CI: confidence interval; HWE: Hardy-Weinberg equilibrium; NA: not applicable.

### Genotypic and allelic frequencies in association analysis

To validate the associations of the five SNPs with HL in the Yugur population, dominant and recessive models for the rare allele were constructed to verify the associations of the variants and to calculate ORs and 95%CIs. Two SNPs were significantly associated with HL in the Yugur population: for *APOB* rs1042034, the AG and AG+AA genotypes were significantly more frequent than the GG genotype, whereas for rs676210, the AG and AG+GG genotypes were significantly more frequent than the AA genotype (Table 2). Thus, the rare alleles of both SNPs in *APOB* were more frequent in HL group than in controls, and were associated with the disease in a dominant manner.

### LD and haplotype analyses

Next, we performed LD and haplotype analyses to explore the associations of multiple SNPs with HL. As shown in Table 3, rs2163204, rs1042034, rs676210, rs679899, rs1042034, and rs676210 were in strong LD. rs2163204, rs512535, rs512535, and rs679899 were also in LD. Haplotype analyses showed a significant difference in the risk for HL between individuals with haplotypes ATGGA and ATAGG and those with haplotype GTGAA (Table 4), suggesting that Yugurs harboring the ATGGA or ATAGG haplotype were more prone to developing HL.

**Table 3.** Standardized linkage disequilibrium coefficients (D′) among five apolipoprotein B gene single nucleotide polymorphisms (SNP).

SNP	rs1042034	rs2163204	rs512535	rs676210	rs679899
rs1042034	–	0.997/0.001*	0.130/0.0001	0.991/0.0001*	0.438/0.0001
rs2163204	-0.197	–	0.806/0.0001*	0.997/0.001*	0.996/0.002*
rs512535	0.127	0.419	–	0.132/0.0001	0.719/0.0001*
rs676210	0.988	-0.197	0.129	–	0.434/0.0001
rs679899	0.354	-0.159	0.568	0.351	–

The upper diagonal values are D′/P, and the lower diagonal values are the correlation coefficients (r).

* Strong linkage disequilibrium.

**Table 4.** Haplotype analyses between the two study groups.

Haplotype	Total frequency (%)	Control frequency (%)	Case frequency (%)	P	OR	95%CI
GTGAA	51.93	50.85	53.57	–		–
ATGGA	14.38	16.30	11.86	0.04	1.46	0.02–2.11
ATAGG	8.28	9.88	6.59	0.04	1.63	1.03–2.60
GGAAA	8.26	7.82	8.46	0.67	0.91	0.59–1.41
GTAAG	7.29	6.40	8.20	0.47	0.84	0.52–1.35
ATGGG	3.17	3.09	3.20	0.84	1.08	0.54–2.13
GTAAA	3.08	2.27	4.05	0.22	0.63	0.30–1.32
GGGAA	1.24	1.50	0.94	0.28	1.85	0.61–5.65
ATAGA	1.10	0.88	1.31	0.55	0.66	0.16–2.62
Rare	1.27	1.01	1.82	0.30	0.54	0.17–1.73

In the order of rs1042034, rs2163204, rs512535, rs676210, and rs679899. OR: odds ratio; CI: confidence interval.

### Assessment of risk factors

We investigated the interactions between genetic variants and other risk factors using one-way analysis and multivariate logistic regression analysis. Specifically, the multivariate logistic regression analysis evaluated the relationship between genetic variants (AG+AA and GA+GG genotypes in rs1042034 and rs676210) and factors for which the P-value was <0.2 in the one-way analysis of variance: age, BMI, SBP, DBP, blood glucose, smoking, and alcohol consumption. Multivariate logistic regression analysis indicated the variables that were ultimately significantly associated with HL in the logistic regression analysis: BMI (P<0.0001), GA+GG genotype in rs676210 (P=0.009), alcohol consumption (P=0.056), and age (P=0.032; Table 5).

**Table 5.** Multivariate regression results of hyperlipidemia pathogenic factors.

Variables	(B)	Wals	P-value	Exp(B)
Age (mean, years)	0.198	4.593	0.032	1.219
BMI (kg/m^2^)	0.570	17.873	<0.0001	1.779
Systolic blood pressure (mmHg)	−0.034	0.036	0.849	0.966
Diastolic blood pressure (mmHg)	0.191	0.458	0.498	1.210
Blood glucose (mg/dL)	0.113	0.184	0.668	1.119
Smoker	−0.199	0.337	0.562	0.819
Drinker	0.369	3.652	0.056	1.446
rs1042034: AG+AA	−0.712	0.330	0.566	0.491
rs676210: GA+GG	0.450	6.865	0.009	1.569

Variables allowed to enter the model were those with P*<*0.20 in the binary logistic regression analysis. BMI: body mass index.

## Discussion

The unique nomadic Chinese Yugur population is of great value for genetic studies. This population has been a target in both etiological and molecular anthropological studies (27,28). Accordingly, in this study, we evaluated five SNPs in the *APOB* gene in middle-aged and elderly members of the Chinese Yugur population, and analyzed the associations of these SNPs with HL. We found that rs1042034 and rs676210 were significantly associated with HL in the Yugur population, and that the two SNPs were in nearly complete linkage. Haplotypes ATGGA and ATAGG were associated with a higher risk of developing HL, and further assessment of risk factors showed that age, BMI, the GA+GG genotype in rs676210, and alcohol consumption were also associated with HL.

Several previous studies have shown that rs1042034, rs676210, and rs512535 in the *APOB* gene promote HL development (29,30 [Bibr B31] [Bibr B32]). In the current study, we found that the SNPs rs1042034 and rs676210 were significantly related to HL in the Chinese Yugur population. Similar results were reported by Kou et al. (33), who showed that rs676210 (AG and AG+GG) in the *APOB* gene was a risk factor for HL in this population. Moreover, in contrast to previous findings (27,29), our data indicated that *APOB* rs512535, rs2163204, and rs679899 had no effect on HL risk in the Chinese Yugur population. The reason for these conflicting results may be related to the small sample size. As mentioned above, the Yugur population is sparse, and due to the inevitable restrictions of the study design, we were only able to recruit 631 subjects for this study. Of course, other possibilities cannot be ruled out, including the influence of the genetic background and living environments of the study participants. Because of their unique living environments and lifestyle habits, nomadic people also exhibit significant differences in individual constitution and disease characteristics compared to other demographic groups, and these differences may be related to specific genetic factors in the Chinese Yugur population.

Linkage SNPs may occur as a whole, without dramatic restructuring of genetic offspring. In our study, the LD and haplotype analyses indicated the presence of strong linkages among *APOB* rs2163204, rs1042034, and rs676210, and strong linkage disequilibrium was observed between rs1042034 and rs676210. The same result was found in the Han Chinese population (34); however, it is not clear whether these SNPs affect the blood lipid levels of the Han Chinese population. Accordingly, Yugur individuals who carry the ATGGA and ATAGG haplotypes may be at a higher risk of developing HL. Although these findings may provide a new approach to improve precision medicine for HL, further studies are needed to confirm these results.

Notably, we found that BMI, the GA+GG genotype in rs676210, alcohol consumption, and age were all associated with HL. Farrall et al. (25) reported an increase in serum TC and serum TGs due to cigarette consumption, regardless of gender, especially for those smoking more than 4 cigarettes a day. However, we did not find a similar effect among the Chinese Yugur, which may be associated with our experimental design, given that we did not classify smokers by the amount and duration of smoking. Our results also showed that high blood pressure and blood sugar had no significant correlation with hyperlipidemia in the Yugur population. A recent study showed that novel diabetes drugs can reduce the risk of cardiovascular events and death (35). Thus, these findings confirmed that genetic variants represent susceptibility factors, which, in cooperation with other common variants and environmental factors, contribute to the development of HL. Similarly, Kim et al. (16) reported that the TC level can be predicted through gene-by-environment interactions between SNPs in the *APOB* gene and dietary cholesterol intake.

In summary, we identified common intronic SNPs (rs676210 and rs1042034) in the *APOB* gene and found five SNPs that were collectively associated with HL in the adult Chinese Yugur population. Importantly, we demonstrated that the G allele of rs676210 may confer an increased risk of HL. In addition, a previous study (36) showed that SNPs in the *APOB* gene are associated with statin pharmacology. Furthermore, HL can be classified into many types, which may be related to the underlying genetic cause. Therefore, determining the genetic background of a patient with HL might be helpful to determine the optimal therapeutic strategy. Our findings may provide insights into the specific role of the *APOB* gene, which may serve as a therapeutic target. Zhou et al. (37) found potential genetic differences between two Yugur subclans (east Yugurs and west Yugurs). Therefore, further studies are needed to examine the functions of these polymorphisms and their implications for the genetics of lipid-lowering drugs, based on the differences between the two Yugur subclans. Moreover, the association between polymorphisms in *APOB* and HL varied according to the gene locus considered. Thus, further studies are also needed to identify more genetic loci and to elucidate their exact functions.
